# Dysregulation of histone modifications in bone marrow mesenchymal stem cells during skeletal ageing: roles and therapeutic prospects

**DOI:** 10.1186/s13287-023-03393-6

**Published:** 2023-06-25

**Authors:** Yujue Li, Mingxing Hu, Jinwei Xie, Shuangqing Li, Lunzhi Dai

**Affiliations:** 1 General Practice Ward/International Medical Center Ward, General Practice Medical Center, National Clinical Research Center for Geriatrics, West China Hospital, Sichuan University, Chengdu, 610041 China; 2 State Key Laboratory of Biotherapy, West China Hospital, Sichuan University, Chengdu, 610041 China; 3 Department of Nuclear Medicine, West China Hospital, Sichuan University, Chengdu, 610041 China; 4 Department of Orthopedics Surgery, West China Hospital, Sichuan University, Chengdu, 610041 China

**Keywords:** BM-MSCs, Methylation, Acetylation, Osteoporosis, Senescence

## Abstract

Age-associated bone diseases such as osteoporosis (OP) are common in the elderly due to skeletal ageing. The process of skeletal ageing can be accelerated by reduced proliferation and osteogenesis of bone marrow mesenchymal stem cells (BM-MSCs). Senescence of BM-MSCs is a main driver of age-associated bone diseases, and the fate of BM-MSCs is tightly regulated by histone modifications, such as methylation and acetylation. Dysregulation of histone modifications in BM-MSCs may activate the genes related to the pathogenesis of skeletal ageing and age-associated bone diseases. Here we summarize the histone methylation and acetylation marks and their regulatory enzymes that affect BM-MSC self-renewal, differentiation and senescence. This review not only describes the critical roles of histone marks in modulating BM-MSC functions, but also underlines the potential of epigenetic enzymes as targets for treating age-associated bone diseases. In the future, more effective therapeutic approaches based on these epigenetic targets will be developed and will benefit elderly individuals with bone diseases, such as OP.

## Introduction

Bone is in a constant dynamic process called bone remodeling, and is involved in a coupling balance between osteoclastic bone resorption and osteoblastic bone formation [1]. Age-associated bone diseases such as osteoporosis (OP) are common in the elderly due to the uncoupling of bone formation and bone resorption [2]. As OP progresses, the bone tissue degenerates and the bone mass decreases, leading to increased susceptibility to fragility fractures [3].Various pathogenic factors, such as ageing [4], alcohol consumption [5], smoking [5], anorexia nervosa [6], concurrent diseases [7, 8], and especially estrogen/androgen deficiency [9], may accelerate the progression of OP. However, estrogen-centric OP pathogenesis has been challenged recently and gradually shifted to ageing-centric OP pathogenesis [10].

Multipotent bone marrow mesenchymal stem cells (BM-MSCs), a class of non-hematopoietic stem cells with the ability to self-renew and differentiate, are the source of pre-osteoblasts essential for bone formation and bone remodeling [11]. Skeletal ageing is a progressive process that involves the inevitable exhaustion and senescence of BM-MSCs and a subsequent decline in bone homeostasis, accompanied by an elevated propensity for increased bone marrow adipose tissue (BMAT) and decreased bone mass [2, 12]. During the ageing process, the self-renewal potential of BM-MSCs is impaired, which manifests in the downregulation of stemness-associated genes such as *Oct4*, *Sox2* and *Nanog*, and the upregulation of senescence-associated genes such as *Cdkn1a* (also known as *p21*, *Cip1*, and *Waf1*), *Cdkn2a* (encoding *p16*^*Ink4a*^ and *p19*^*Arf*^ in mice and *p14*^*Arf*^ in humans) and *Cdkn2b* (encoding *p14*^*Ink4b*^ and *p15*^*Ink4b*^) [13–15]. Senescence of BM-MSCs, including the dysregulation of BM-MSC lineage commitment in the senescent bone marrow microenvironment, is critical to the occurrence of OP [16, 17]. Senescent BM-MSCs accumulate in the bone marrow with ageing, characterized by reduced proliferation, enhanced adipogenesis and decreased osteogenesis, and may lead to bone marrow adiposity, bone loss and increased risk of major fractures [2, 4].

Histone modifications are important regulators of the lineage commitment and senescent process of BM-MSCs and control the process of skeletal ageing [15, 18–25]. Here, we summarize the latest findings that histone methylation and acetylation regulate the senescence, self-renewal and differentiation of BM-MSCs during bone ageing, and highlight the potential of regulatory enzymes as therapeutic targets for age-associated diseases, such as OP.

## Histone modifications

The impaired function of senescent stem cells is often accompanied by changes in epigenetic modifications, such as DNA methylation, histone alteration, chromatin remodeling, m^6^A modulation and ncRNA-mediated regulation of gene expression [26, 27]. Histone modifications and their corresponding regulatory enzymes cause chromatin remodeling without altering the primary DNA sequence, serving as critical modulators in lineage commitment and the senescent process of BM-MSCs [20, 28–30]. Methylation, acetylation, phosphorylation, ubiquitination and sumoylation are well-known covalent histone modifications that take place on active residues in histones that are crucial for chromatin architecture, nucleosome stability and gene transcription [31, 32]. These histone modifications not only alter the histone-DNA binding affinity, but also influence chromatin compaction and accessibility, which results in changes in the folding or exposure state of target gene promoters and affects gene expression [32–34].

**Fig. 1:**
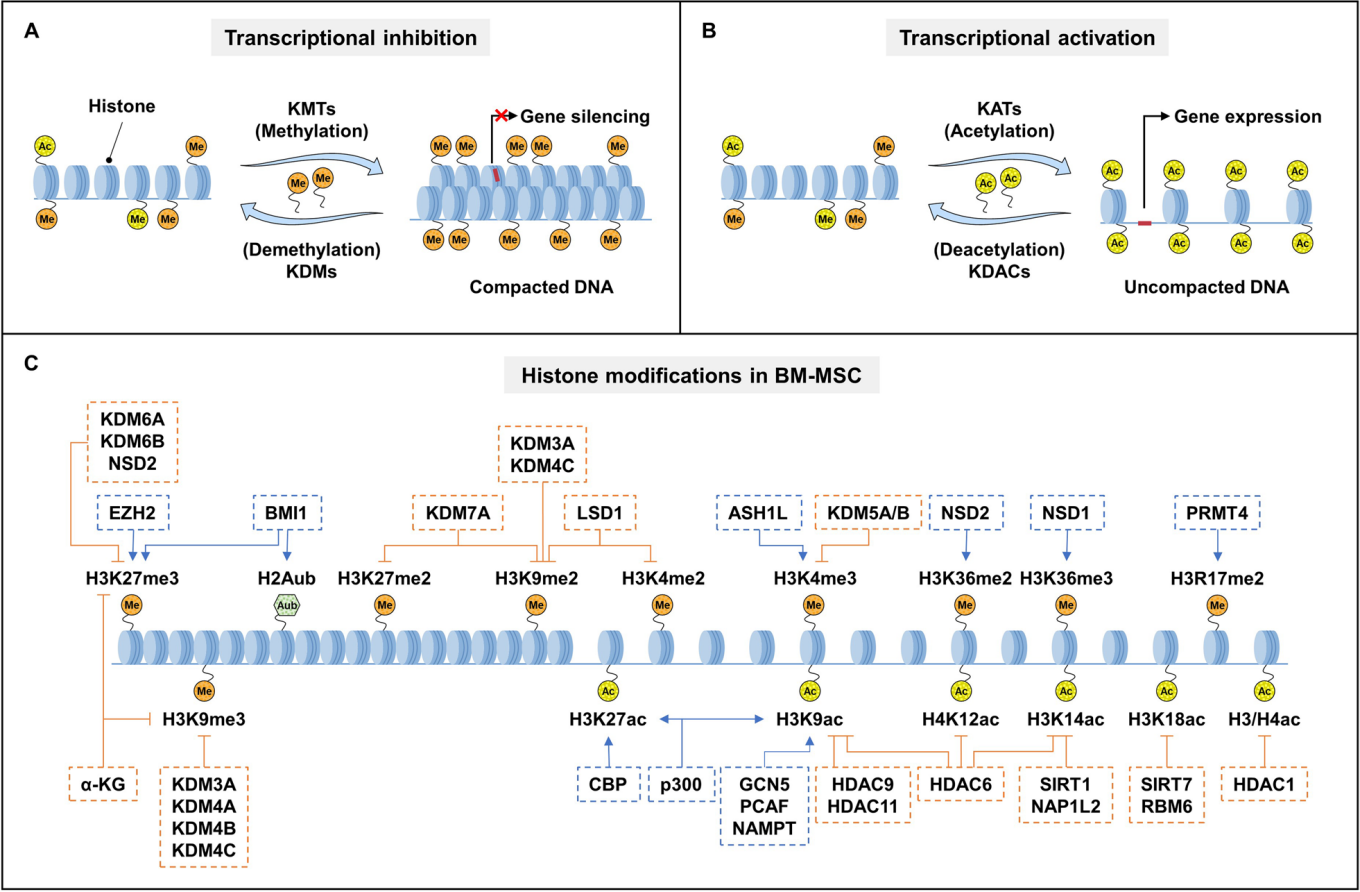
The landscape of histone modifications in the regulation of BM-MSCs.** A** The schematic diagram of histone lysine methylation. **B** The schematic diagram of histone lysine acetylation. **C** Histone modifications in BM-MSC during skeletal ageing

Methylation and acetylation are the most widely studied histone modifications (Fig. 1A, B). Histone methylation typically occurs on lysine (*K*) (including mono-, di- and trimethylation) and arginine (*R*) (monomethylation, and symmetric or asymmetric dimethylation) residues mediated by histone methyltransferases (HMTs) and can be removed by demethylases (HDMs) [35, 36]. In general, methylation at H3K4, H3K36, H3K79 and H3R17 promotes transcriptional activation, whereas methylation at H3K9, H3K27 and H4K20 tends to repress transcription [37, 38]. For instance, an increased level of H3K27me3 often indicates a tighter and repressive state of nucleosomes linked to gene silencing [39]. Similarly, lysine acetylation is a dynamic modification that can be added by lysine acetyltransferases (KATs) and removed by lysine deacetylases (KDACs) [40]. KAT-mediated lysine acetylation causes loose chromatin and transcriptional activation. Deacetylation by KDACs causes chromatin condensation leading to gene silencing [41, 42]. These diverse histone modifications constitute a network that regulates the fate of BM-MSCs (Fig. 1C) [43–45].

## Regulation and functions of histone modifications in BM-MSCs

### Lysine methylation

**Fig. 2:**
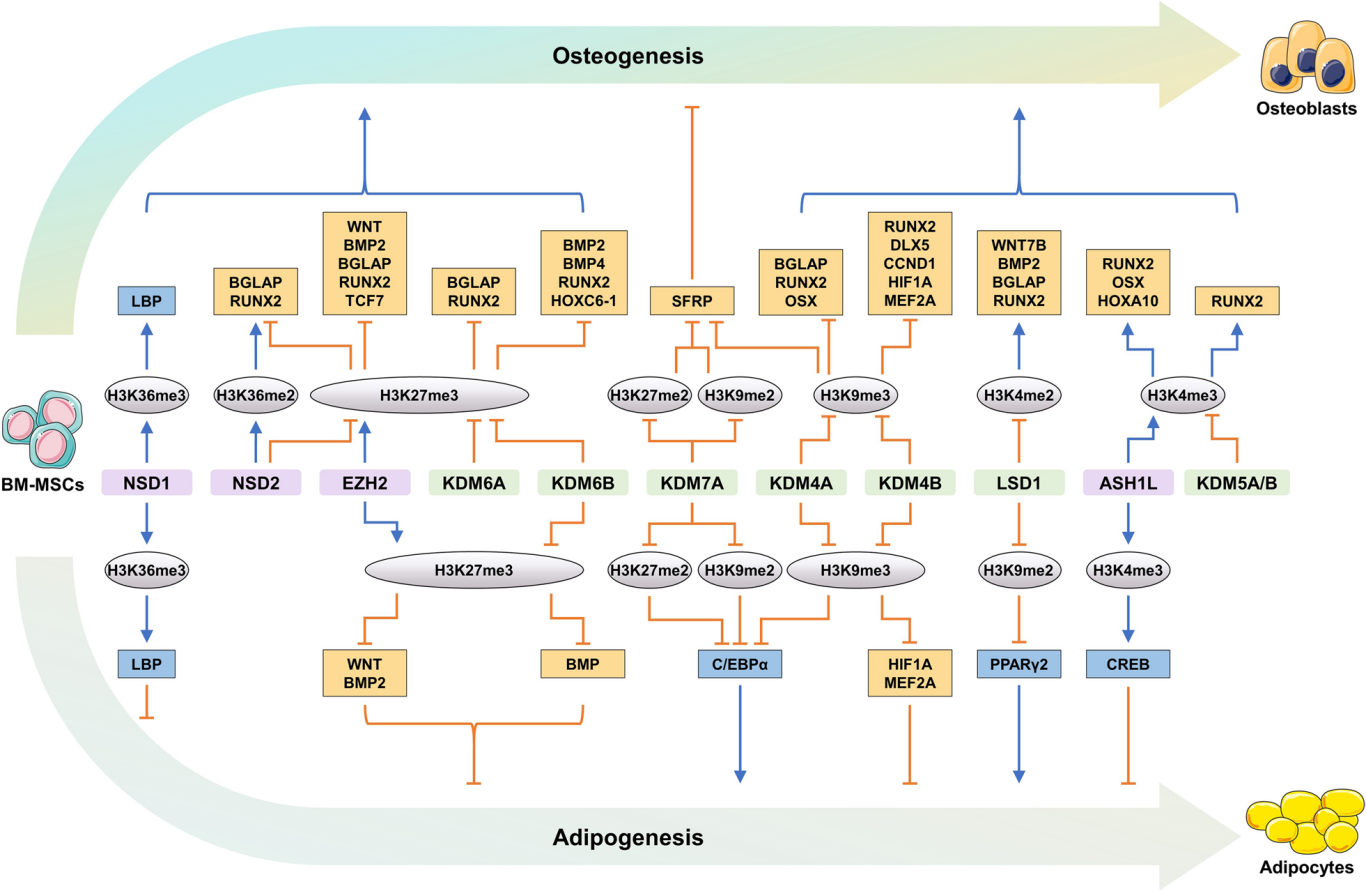
KMTs and KDMs regulate the osteogenic and adipogenic differentiation of BM-MSCs by histone methylation

Lysine methylation is a well-understood epigenetic mechanism in BM-MSC fate regulation mediated by histone lysine methyltransferases (KMTs) and demethylases (KDMs) (Fig. 2 and Table 1) [43]. The KMTs, including DOT1L and SET domain-containing proteins, are responsible for methylation at K4, K9, K27, K36 and K79 of H3, as well as K20 of H4. In contrast, apart from LSD1, all known KDMs have a conserved JmjC domain. Therefore, KDMs are also termed JmjC domain-containing histone demethylases (JHDMs) [36]. KMTs and KDMs reversibly and dynamically regulate methylation at lysine residues of histones, thus modulating the transcription of target genes.

**Table 1:** Histone methylation and related modifiers regulate the fate of BM-MSC

Modifiers	Histone modification	Targets	Effects on fate of BM-MSC (in vitro)	Effects on bone (in vivo)	References
*Methylases*					
ASH1L	H3K4me3	*Osx*, *Runx2*, *Hoxa10*, *Sox9*, *Creb*	Promotes osteogenesis and chondrogenesis, while inhibiting adipogenesis	Not evaluated	[70]
EZH2 (KMT6A)	H3K27me3	Osteoblastic genes (*RUNX2*, *TCF7*, *BGLAP*, *Wnt*, *Bmp2*), senescence-associated genes (*p14*^*Arf*^, *p16*^*Ink4a*^, *p21*^*Cip1*^), antioxidant *Foxo1*	Inhibits osteogenesis, while promoting adipogenesis, restraining senescence, accumulating oxidative damage	Inconsistent conclusion	[14, 15, 19, 28, 47, 50, 61]
NSD1 (SETD2, SET2, KMT3A)	H3K36me3	*Lbp*	Promotes osteogenesis while inhibiting adipogenesis	Promotes bone mass increase	[29]
NSD2 (MMSET)	H3K36me2 H3K27me3	*Runx2*, *Bglap*	Promotes osteogenesis while inhibiting senescence	Not evaluated	[21]
SETD7 (SET7, SET9, SET7/9, KMT7)	H3K4me3	Not evaluated	Promotes osteogenesis	Not evaluated	[72]
PRMT3	H4R3me2a	miR-3648	Promotes osteogenesis	Promotes bone regeneration and bone mass increase	[103]
PRMT4 (CARM1)	H3R17me2	*OCT4*, *SOX2*, *NANOG*, *DDR2*	Promotes pluripotency while inhibiting senescence	Not evaluated	[104, 105]
*Demethylases*					
LSD1 (KDM1A)	H3K9me2	*Pparγ2*	Promotes adipogenesis	Not evaluated	[64]
LSD1	H3K4me2	*Runx2*, *Bglap*, *Wnt7b*, *Bmp2*	Inhibits osteogenesis	Promotes bone loss	[73, 74]
KDM3A (JMJD1A, JHDM2A)	H3K9me2 H3K9me3	*Ncapd2*, *Ncapg2*	Inhibits DNA damage and senescence	Inhibits bone loss	[68]
KDM4A (JMJD2A, JHDM3A, JMJD2)	H3K9me3	*Runx2*, *Osx*, *Bglap*	Promotes osteogenesis	Not evaluated	[66]
KDM4A	H3K9me3	*Sfrp4*, *C/ebpα*	Promotes adipogenesis while inhibiting canonical Wnt signaling	Not evaluated	[65]
KDM4B (JMJD2B, JHDM3B)	H3K9me3	*Runx2*, *Dlx5*, *Ccnd1*, *Hif1α*, *Mef2a*	Promotes osteogenesis while inhibiting adipogenesis	Inhibits age-related bone loss	[18, 54]
KDM4C (GASC1, JMJD2C, JHDM3C)	H3K9me2 H3K9me3	*Ncapd2*, *Ncapg2*	Inhibits DNA damage and senescence	Inhibits bone loss	[68]
KDM5A (JARID1A, RBP2)	H3K4me3	*Runx2*	Inhibits osteogenesis	Promotes bone loss	[75]
KDM5B (JARID1B, PLU1)	H3K4me3	*Runx2*	Inhibits osteogenesis	Not evaluated	[76]
KDM6A (UTX)	H3K27me3	*RUNX2*, *BGLAP*	Promotes osteogenesis	Not evaluated	[47, 52]
KDM6B (JMJD3)	H3K27me3	*Bmp2*, *Bmp4*, *Hoxc6-1*, *Runx2*	Promotes osteogenesis while inhibiting adipogenesis	Not evaluated	[54, 55]
KDM7A (KIA1718, JHDM1D)	H3K9me2 H3K27me2	*C/ebpα* *Sfrp1*	Promotes adipogenesis while inhibiting osteogenesis	Not evaluated	[58]
*Other regulators*					
BMI1	H2Aub H3K27me3	*p14*^*Arf*^, *p16*^*Ink4a*^, *Pax3*	Promotes hematopoiesis while inhibiting adipogenesis and senescence	Inhibits bone marrow adiposity	[62]
α-KG	H3K9me3 H3K27me3	*Bmp2*, *Bmp4*, *Nanog*	Promotes proliferation, migration and osteogenesis	Inhibits age-related bone loss and promotes bone defect healing	[20]
NO66	H3K4me3 H3K36me3	Not evaluated	Inhibits osteogenesis	Inhibits endochondral and intramembranous bone formation	[81]

Not evaluated: the effects of histone modification enzymes or related modifiers on bone were not verified in vivo

#### H3K27 methylation

**Fig. 3:**
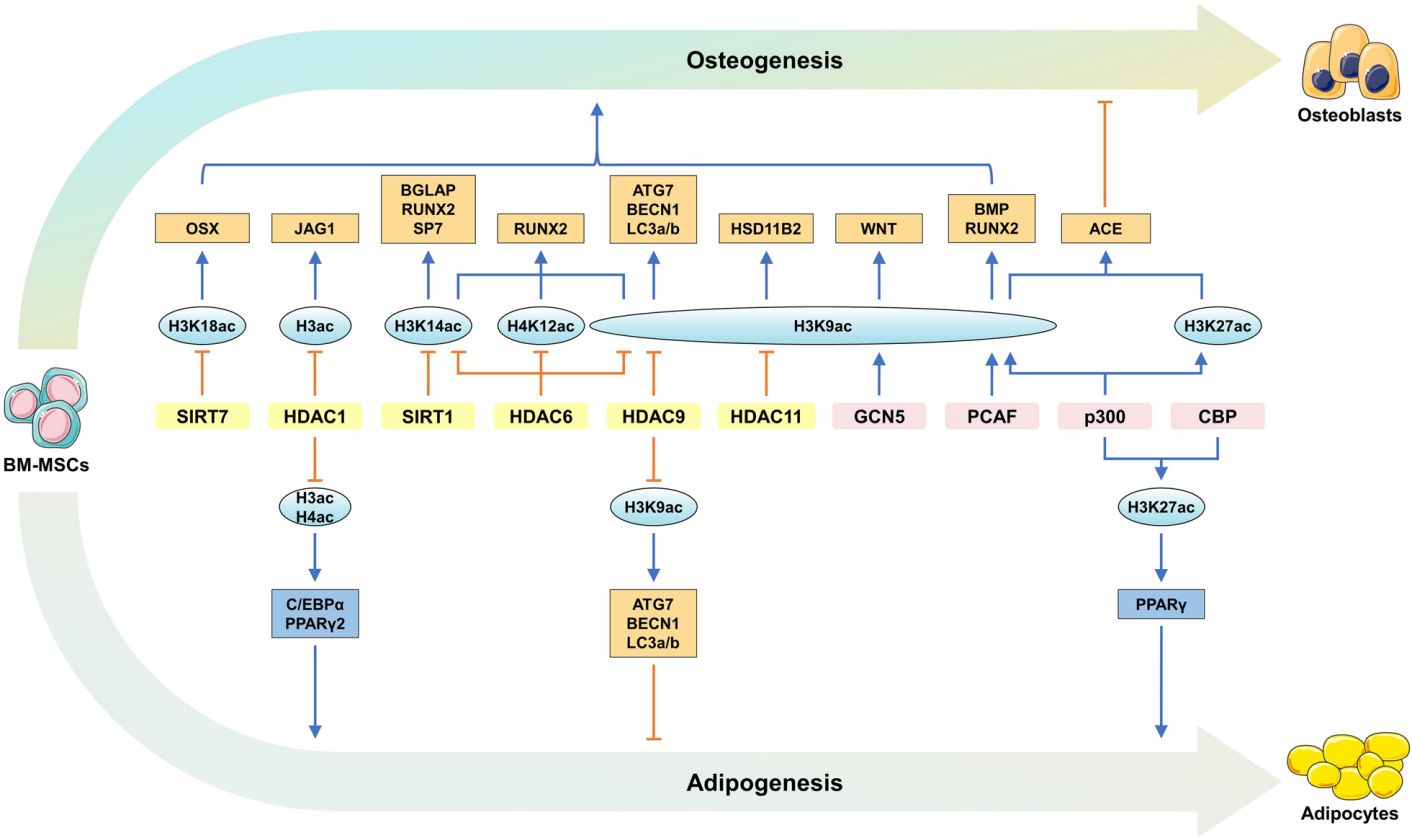
KATs and KDACs regulate the osteogenic and adipogenic differentiation of BM-MSCs by histone acetylation

Methylation at H3K27 acts as an important epigenetic switch dictating BM-MSC lineage determination (Fig. 2 and Table 1). Elevated H3K27me3 on pro-osteogenic gene promoters inhibits osteogenesis of BM-MSCs, while H3K27me2 on anti-osteogenic gene promoters impedes adipogenesis of BM-MSCs. EZH2 (also termed KMT6A) catalyzes the methylation of H3K27 on target gene promoters [39]. EZH2 acts as a negative regulator of osteogenesis by increasing H3K27me3 on the promoters of osteoblastic genes like *RUNX2*, *TCF7* and *BGLAP *in vitro [46, 47]. EZH2 is significantly elevated in osteoporotic BM-MSCs and directly upregulates H3K27me3 levels on the promoters of *Wnt1*, *Wnt6*, *Wnt10a* and *Wnt10b* to impede *Wnt* gene transcription [28, 48]. The inhibition of Wnt/β-catenin signaling shifts MSC lineage commitment to adipocyte during OP [28]. *Ezh2* deletion upregulates *Bmp2*, *Runx2* and *Wnt* expression, and accelerates bone remodeling [49, 50]. The methylation state of H3K27 is dynamically regulated by the EZH2 and KDM6 cluster. The KDM6 cluster contains three members, including KDM6A (also termed UTX), KDM6B (also called JMJD3) and inactive UTY [51]. KDM6A and KDM6B are positive regulators of osteogenesis by removing the methyl groups of H3K27 on osteogenic genes. For example, KDM6A demethylates H3K27me3 on osteogenic genes (e.g., *Runx2* and *Bglap*) and activates the expression of these genes in human and mouse BM-MSCs [47, 52, 53]. KDM6B demethylates H3K27me3 to promote the expression of *Bmp2*, *Bmp4*, *Runx2* and *Hoxc6-1* and induce osteogenic commitment of BM-MSCs, thus elevating bone mass in OVX and aged mice [54, 55]. Similar results have also been demonstrated in human dental MSCs [56]. KDM7A (also called KIAA1718 or JHDM1D) has demethylase activity for H3K27me1/me2 and H3K9me1/me2 [57], and can enhance adipogenesis and weaken osteogenesis by demethylating H3K9me2 and H3K27me2 on the promoters of *Sfrp1* and *C/ebpα* in mouse primary BM-MSCs and ST2 cells [58]. Alpha-ketoglutarate (α-KG), an essential endogenous metabolite in the tricarboxylic acid (TCA) cycle, is reported to extend lifespan and compress morbidity in ageing mice [59, 60]. Alpha-KG treatment reduces H3K27me3 at the *Bmp2*, *Bmp4* and *Nanog* promoters, thus restoring the proliferation, migration and osteogenesis abilities of aged BM-MSCs [20]. Collectively, H3K27me3 on pro-osteogenic gene promoters is mainly regulated by EZH2, the KDM6 cluster and α-KG, whereas H3K27me2 on anti-osteogenic genes is partially affected by KDM7A (Fig. 3).

**Fig. 4:**
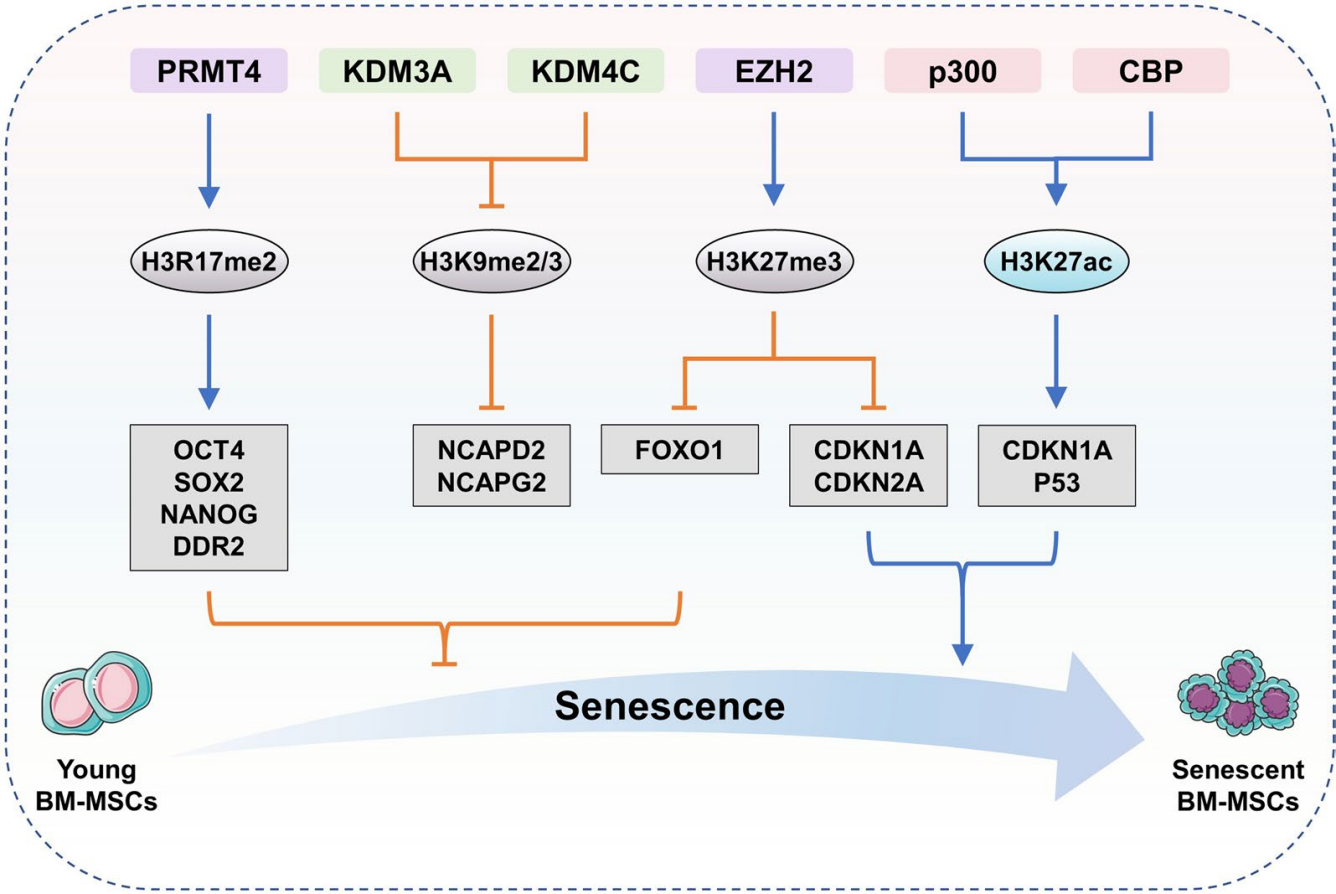
Histone modifications regulate BM-MSCs senescence

As a regulatory center for lineage determination of BM-MSCs, H3K27 methylation plays an important role in regulating cellular senescence (Fig. 4 and Table 1). EZH2 upregulates the repressive mark H3K27me3 at the promoters of cell cycle inhibitor genes (e.g., *p14*^*Arf*^, *p16*^*Ink4a*^ and *p21*^*Cip1*^), and loss of EZH2 results in transcriptional activation of these genes to promote senescence of BM-MSCs [14, 15, 61]. However, EZH2 enhances H3K27me3 in the promotor of *Foxo1* to inactivate the antioxidative defensive system, thus promoting oxidative damage and BM-MSC ageing [19]. Thus, EZH2 shows bifunctional roles in regulating BM-MSC senescence. Notably, BMI1 can prevent senescence and adipogenesis of BM-MSCs by increasing H3K27me3 and H2A ubiquitination (H2Aub) of *p14*^*Arf*^, *p16*^*Ink4a*^ and *Pax3* [62]. Therefore, EZH2 and BMI1 jointly regulate the ageing process of BM-MSCs.

#### H3K9 methylation

H3K9 methylation on the promoters of adipogenic *Pparγ2* and *C/ebpα*, as well as anti-osteogenic *Sfrp* inhibits the transcription of these genes, which consequently impedes adipogenesis and promotes osteogenesis of BM-MSCs (Fig. 2 and Table 1). KDMs are key components of potent epigenetic switches that control BM-MSC fates into adipogenic lineages. LSD1 (also termed KDM1A) regulates gene transcription by demethylating H3K9me1/me2 and H3K4me1/me2 [63]. By demethylating H3K9me2, LSD1 induces *Pparγ2* gene expression and promotes adipogenic differentiation of BM-MSCs [64]. The KDM4 cluster is widely expressed in human tissues and can demethylate H3K9me2/me3 and H3K36me2/me3 [43]. KDM4A (also known as JMJD2A, JHDM3A and JMJD2) activates *C/ebpα* and *Sfrp4* transcription by demethylating H3K9me3, thus promoting adipocyte formation and inhibiting bone formation in mouse primary BM-MSCs and ST2 cells [65]. As mentioned above, the removal of H3K9me2 and H3K27me2 by KDM7A also shows similar functions [58]. Taken together, LSD1, KDM4A and KDM7A play a negative role in bone formation through demethylating H3K9me2/me3 at the promoters of adipogenic genes (e.g., *Pparγ2* and *C/ebpα*) and anti-osteogenic genes (e.g., *Sfrp*).

H3K9 methylation can repress the expression of pro-osteogenic genes (e.g., *Bmp2*, *Runx2*, *Osx*, *Bglap*, *Dlx5*, *Ccnd1*, *Hif1α*, *Mef2a* and *Nanog*), thereby inhibiting osteogenic differentiation of BM-MSCs (Fig. 2 and Table 1). The demethylases KDM4A and KDM4B (also termed JMJD2B and JHDM3B) show crucial and positive functions in the osteogenic differentiation of BM-MSCs. KDM4A promotes osteoblast differentiation of rat primary BM-MSCs by removing the silencing epigenetic mark H3K9me3 on osteoblastic genes (*Runx2*, *Osx* and *Bglap*) [66]. Similarly, KDM4B significantly upregulates pro-osteogenic gene expression (e.g., *Runx2*, *Dlx5*, *Ccnd1*, *Hif1α* and *Mef2a*) by demethylating repressive H3K9me3 on the promoters of these genes [18, 54]. Treatment of aged BM-MSCs with α-KG downregulates H3K9me3 occupancy at the *Bmp2* and *Nanog* promoters, ultimately promoting proliferation and osteogenesis of aged BM-MSCs [20]. Therefore, KDM4A, KDM4B and α-KG have positive functions in alleviating skeletal ageing by removing the repressive H3K9me3 on osteoblastic genes to strengthen osteogenesis.

H3K9 demethylases KDM3A (also called JMJD1A and JHDM2A) and KDM4C (also known as GASC1, JMJD2C and JHDM3C) are negatively correlated with BM-MSC senescence [36, 43]. H3K9 methylation along with heterochromatin loss drives human MSC ageing [67]. KDM3A and KDM4C remove the methyl groups of H3K9me2/me3 on the promoters of *NCAPD2* and *NCAPG2* to activate them, which restrains the accumulation of damaged DNA through inducing heterochromatin reorganization, suggesting the protective roles of demethylated H3K9 in BM-MSC senescence and bone ageing (Fig. 4 and Table 1) [68].

Collectively, methylated H3K9 on pro-osteogenic genes is strongly dependent on the levels of KDM3A, the KDM4 cluster and α-KG, whereas H3K9me2/me3 on anti-osteogenic genes is affected by LSD1, KDM4A and KDM7A.

#### H3K4 methylation

Elevated H3K4 methylation can promote osteogenesis (Fig. 2 and Table 1). ASH1L, a member of the Trx family, activates the expression of multiple genes via its H3K4 and H3K36 methyltransferase activity of the SET domain [36]. ASH1L and H3K4me3 bind to the transcription start site (TSS) of *Hoxa10*, *Osx*, *Runx2*, *Sox9* and *Creb*. Out of them, SOX9 is an important transcription factor that promotes cartilage formation, and CREB may act as a repressive gene of PPARγ [69, 70]. ASH1L interference downregulates H3K4me3 at the TSS of these genes, which inhibits osteogenesis and chondrogenesis and promotes adipogenesis [70]. Another SET domain-containing protein SETD7 (also termed KMT7, SET7, SET9 and SET7/9) is also a methyltransferase of H3K4 [71]. The trace element boron promotes bone regeneration in vivo and stimulates the osteogenic differentiation of human BM-MSCs in vitro by increasing SETD7 and successive H3K4me3, which may further activate the Wnt/β-catenin pathway [72]. Collectively, ASH1L and SETD7 are two methylases associated with the promotion of bone formation by methylating H3K4.

Notably, the LSD1 and KDM5 cluster are demethylases that inhibit osteogenic differentiation by removing the methyl groups of H3K4me2/me3 on osteoblastic gene promoters. LSD1 inhibition rescues the osteogenic differentiation ability of BM-MSCs in OVX mice by enhancing H3K4me2 on the promoters of osteogenic genes (e.g., *Runx2* and *Bglap*) [73]. In addition, LSD1 deficiency results in H3K4me2 enrichment on *Wnt7b* and *Bmp2* promoters and enhances bone formation in *Prx1-Cre;Lsd1*^*fl/fl*^ mice [74]. KDM5A (also termed JARID1A and RBP2) and KDM5B (also called JARID1B and PLU1) can catalyze the removal of mono-, di-, and tri-methyl marks on H3K4 to regulate gene expression [36]. KDM5A and KDM5B repress osteogenesis by downregulating H3K4me3 on the promoter of *Runx2* gene [75, 76]. Taken together, the results suggest that the demethylases LSD1 and KDM5 cluster inhibit osteogenesis by demethylating H3K4me2/me3 on the promoters of osteogenic genes, including *Runx2*, *Bglap*, *Wnt7b* and *Bmp2*.

#### H3K36 methylation

Methylation of H3K36 on *Sox9*, *Lbp*, *Runx2*, and *Bglap* genes promotes chondrogenesis, inhibits adipogenesis, and accelerates osteogenesis (Fig. 2 and Table 1). NSD1 (also termed SETD2, SET2 and KMT3A) and NSD2 (also called MMSET) are well-known H3K36 methyltransferases [36, 77]. Deletion of *Nsd1* decreases *Sox9* expression by reducing H3K36me1/me2 levels, leading to chondrogenic differentiation impairment [78]. The transcription initiation and elongation of the *Lbp* gene is maintained by NSD1-induced H3K36me3 in BM-MSCs [29]. LBP negatively regulates adipocyte differentiation and contributes to a decreased propensity toward adipogenesis and an elevation in bone formation [79]. NSD2-mediated upregulation of H3K36me2 and downregulation of H3K27me3 can increase chromatin accessibility and facilitate osteogenic gene expression (*Runx2* and *Bglap*), consequently ameliorating age-associated bone loss [21]. NO66 has been identified as a JmjC-containing oxygenase, with demethylase activity for methylated H3K4 and H3K36 [36], and can inhibit endochondral and intramembranous bone formation during skeletal development [80, 81]. Therefore, NSD1, NSD2 and NO66 regulate the fate of BM-MSCs by modulating H3K36 methylation.

### Lysine acetylation

Reversible protein lysine acetylation is mediated by KATs and KDACs (Fig. 1B) [42]. Mammalian KATs are classified into type A KATs localized in the nucleus and type B KATs present in the cytoplasm. Nuclear histone acetylation events regulated by Type A KATs are usually involved in transcriptional activation [41, 42]. Type A KATs are divided into five families, including GNATs, p300/CBP, MYST (MOZ, YBF2, SAS2 and TIP60), basal transcription factors, and nuclear receptor coactivator (NCoA) subfamilies. In contrast, the number of type B KATs is much smaller. Type B KATs acetylate free histones in the cytoplasm and facilitate the transport of cytosolic histones into the nucleus [82]. Based on sequence homology and domain organization, KDACs are classified into four groups. Classes I, II and IV belong to Zn^2+^-dependent histone deacetylases (HDACs), whereas class III KDACs are characterized as NAD^+^-dependent sirtuins (SIRTs) including SIRT1 to SIRT7 [42, 83].

Histone acetylation is generally associated with the opening of the chromatin structure and enhanced transcriptional activity, which are closely linked to bone homeostasis (Fig. 3 and Table 2). However, KDACs appear to be less selective for histones in regulating BM-MSC fates, as the vast majority of KDACs are ‘pan’ deacetylases. For example, reduced HDAC1 induces hyperacetylation of H3 and H4 on promoters of adipogenic genes (e.g., *Pparγ2* and *C/ebpα*) in BM-MSCs of GIOP mice [64, 84]. Here we will focus on the effect of H3K9ac, H3K14ac, H3K27ac and H3K18ac on the regulation of BM-MSC functions.

**Table 2:** Histone acetylation and related modifiers regulate the fate of BM-MSC

Modifiers	Histone modification	Targets	Effects on fate of BM-MSC (in vitro)	Effects on bone (in vivo)	References
*Acetylases*					
GCN5 (KAT2A)	H3K9ac	*Vegf*,* Wnt*	Promotes angiogenesis and osteogenesis	Inhibits bone loss	[85, 88]
PCAF (KAT2B)	H3K9ac	*BMP2*, *BMP4*, *BMPR1B*, *RUNX2*	Promotes osteogenesis	Not evaluated	[86]
p300 (KAT3B)	H3K9ac H3K27ac	*α-KG*	Inhibits osteogenesis	Not evaluated	[98, 99]
p300/CBP	H3K27ac	*Pparγ*, *p53*, *p21*^*Cip1*^	Promotes adipogenesis and senescence	Not evaluated	[100]
*Deacetylases*					
HDAC1	H3/H4ac	*Pparγ2*, *C/ebpα*	Inhibits adipogenesis	Not evaluated	[64, 84]
HDAC1	H3ac	*Jag1*	Inhibits osteogenesis	Promotes bone loss	[109]
HDAC6	H3K9/K14ac H4K12ac	*Runx2*	Inhibits osteogenesis	Promotes age-related bone loss	[91]
HDAC9	H3K9ac	*Atg7* *Becn1* *LC3a/b*	Promotes adipogenesis while inhibiting autophagy and osteogenesis	Promotes age-related bone loss	[92]
HDAC11	H3K9ac	*Hsd11b2*	Inhibits osteogenesis	Not evaluated	[93]
SIRT1	H3K14ac	*Runx2* *Sp7* *Bglap*	Inhibits osteogenesis	Not evaluated	[94]
SIRT7	H3K18ac	*OSX*	Inhibits osteogenesis	Not evaluated	[101]
*Other regulators*					
NAMPT	H3K9ac	*Runx2*	Promotes osteogenesis	Not evaluated	[87]

Not evaluated: the effects of histone modification enzymes or related modifiers on bone were not verified in vivo

#### H3K9 acetylation

Upregulated acetylation of H3K9 on the promoters of osteogenic genes, such as *WNT, BMP* and *RUNX2*, has crucial roles in the osteogenic commitment of BM-MSCs (Fig. 3 and Table 2) [85–87]. Downregulated H3K9ac on the *Wnt* gene in BM-MSCs from OVX mice leads to persistent suppression of WNT signaling. Overexpression of GCN5 (also known as KAT2A) promotes osteogenic differentiation of BM-MSCs by increasing H3K9ac on the promoters of *Wnt* genes (*Wnt1*, *Wnt6*, *Wnt10a*, and *Wnt10b*) [85]. GCN5 enhances the proangiogenesis of BM-MSCs by increasing H3K9ac levels on the *Vegf* promoter, consequently contributing to bone formation [88]. In addition, GCN5 can inhibit anti-osteogenic NF-κB signaling by degrading the p65 subunit of NF-κB [89]. PCAF (also known as KAT2B) promotes osteogenic differentiation by catalyzing the acetylation of H3K9 on *BMP2*, *BMP4*, *BMPR1B* and *RUNX2* promoters [86]. In the salvage pathway, NAMPT acts as one of the most critical enzymes controlling NAD^+^ biosynthesis from nicotinamide [90]. The transcription of *Runx2* can be enhanced due to a NAMPT-associated increase in H3K9ac [87]. Collectively, GCN5, PCAF and NAMPT enhance the osteogenic capacity of BM-MSCs mainly by upregulating the level of H3K9ac on osteogenic gene promoters.

HDACs are deacetylases of H3K9ac, by which HDACs inhibit the osteogenic differentiation potential of BM-MSCs (Fig. 3 and Table 2). For example, HDAC6 accumulation and histone hypoacetylation, including H3K9/K14ac and H4K12ac, on the *Runx2* promoter contribute to the attenuation of the osteogenic differentiation potential of BM-MSCs in aged mice [91]. HDAC9 directly represses the transcription of genes related to autophagy, such as *Atg7*, *LC3a/b* and *Becn1*, and impairs the autophagy of BM-MSCs by deacetylating H3K9ac, which causes a shift of cell lineages from osteoblasts to adipocytes and leads to skeletal ageing [92]. Prenatal dexamethasone exposure recruits HDAC11 into the nucleus and reduces the expression of *Hsd11b2* by deacetylating H3K9ac, which lasts into adulthood and causes corticosterone accumulation in bone. This condition persisting into adulthood will inhibit the osteogenic function of BM-MSCs [93]. Collectively, HDAC 6, 9, and 11 can be able to inhibit BM-MSCs osteogenesis by deacetylating H3K9ac on the promoters of *Runx2*, *Hsd11b2* and autophagy-related genes.

#### H3K14 acetylation

Increased H3K14ac on the promoters of genes, including *Runx2, Sp7, Bglap* and *Igf1*, can promote the osteogenesis (Fig. 3 and Table 2) [91, 94, 95]. HDAC6 deacetylates H3K14ac on the *Runx2* promoter and attenuates osteogenic differentiation potential of BM-MSCs in aged mice [91]. Elevated NAP1L2, a histone chaperone, reduces the level of H3K14ac by recruiting SIRT1, thereby preventing osteogenic gene expression (e.g., *Runx2*, *Sp7* and *Bglap*) and inhibiting osteogenic differentiation of MSCs [94]. The enrichment of H3K9ac and H3K14ac at the *Igf1* promoter upregulates the expression of IGF1 in liver and IGF1 signaling in bone, which promotes bone development and bone mass increase [95, 96]. In addition, the increase in H3K9ac and H3K14ac is also correlated with a decreased HDAC1 level [96]. Collectively, HDAC1, HDAC6 and SIRT1 inhibit osteogenesis by deacetylating H3K14ac on pro-osteogenic gene promoters, including *Runx2, Sp7, Bglap* and *Igf1*.

#### H3K27 acetylation

Elevated H3K27ac, mediated by CBP (also termed KAT3A) and p300 (also known as KAT3B) [97], can inhibit osteogenesis by increasing the levels of ACE, PPARγ, ageing-related p53 and p21^Cip1^ (Figs. 3 and 4, Table 2) [98–100]. Dexamethasone or ethanol exposure during pregnancy upregulates H3K27ac of *Ace* and its expression by recruiting p300, which further induces sustained activation of renin-angiotensin systems (RAS) and suppresses osteogenic differentiation of BM-MSCs, thereby leading to fetal bone development inhibition and osteopenia after birth [98, 99]. P300/CBP activation by maternal obesity results in H3K27ac on the promoters of the *Pparγ*, *p53* and *p21*^*Cip1*^ genes in mouse embryonic calvarial osteo-progenitors and in human umbilical cord MSCs, suggesting that obesity during pregnancy may impair osteogenesis in adult offspring [100]. Collectively, p300/CBP inhibits osteogenesis via H3K27ac on the promoters of anti-osteogenic genes, including *Ace*, *Pparγ*, *p53* and *p21*^*Cip1*^.

#### H3K18 acetylation

Acetylation of H3K18 on the osteogenic *OSX* gene can promote osteoblast differentiation of BM-MSCs (Fig. 3 and Table 2). RBM6 recruits SIRT7 to deacetylate H3K18ac and inhibit the expression of isoforms 1 and 2 of the *OSX* gene [101]. In addition, SIRT7 can also repress osteogenesis of human BM-MSCs partially by inactivating the Wnt/β-catenin pathway [102]. Therefore, SIRT7 appears to be a potential therapeutic target for OP.

### Arginine methylation

Protein arginine methyltransferases (PRMTs) are divided into 3 subcategories: type I (including PRMT1, 2, 3, 4, 6 and 8), type II (including PRMT5 and 9) as well as type III (only PRMT7) PRMTs [36]. Compared to lysine methylation in BM-MSCs, the regulation and functions of arginine methylation in BM-MSCs are relatively less studied but very important. For example, PRMT3 is an arginine methyltransferase responsible for catalyzing ω-mono- or asymmetric dimethylation on arginine. The expression of miR-3648 is increased by elevating H4 arginine 3 asymmetric dimethylation (H4R3me2a), consequently leading to increased osteogenic differentiation of BM-MSCs [103]. PRMT4 (also termed CARM1) can induce the expression of *OCT4*, *SOX2* and *NANOG* by upregulating H3R17me2 on the promoters of stemness-associated genes, thereby enhancing the adipogenic, osteogenic and myogenic differentiation potentials of human BM-MSCs and adipose-derived MSCs [104]. In addition, PRMT4 is capable of binding to the *DDR2* promoter region and upregulates H3R17me2 in vitro, which can enhance *DDR2* expression and restrain cellular senescence [105]. Collectively, PRMT3 and PRMT4 promote osteogenesis by increasing H4R3me2a on miR-3648 and H3R17me2 on *OCT4*, *SOX2*, *NANOG* and *DDR2* gene promoters (Fig. 4 and Table 1).

## Histone modification enzymes are potential targets for OP

Impaired proliferation and biased differentiation of BM-MSCs lead to decreased bone homeostasis, a hallmark of skeletal ageing, with a tendency to increase BMAT and decrease bone mass [2, 12]. Histone modifications are critical for regulating the fate and functions of BM-MSCs, and a large number of preclinical studies suggested that histone modification enzymes could serve as potential targets for enhancing bone formation and treating OP. Small molecule inhibitors of histone modification enzymes such as EZH2, LSD1, and HDAC have been applied to treat hematological cancers in the clinic [106]. Accumulating findings suggest that the modulation of histone modifications can be used to improve osteogenic differentiation of BM-MSCs, increase bone strength, and prevent skeletal ageing. For example, EZH2 acts as a negative regulator of osteogenesis by increasing H3K27me3 on osteoblastic genes and inhibits the osteogenic lineages of BM-MSCs [28, 46–48]. Therefore, EZH2 inhibitors have osteoprotective potential and offer an opportunity for bone anabolic strategies [107, 108]. Estrogen is an important medication for postmenopausal osteoporosis (PMOP) and can induce the expression of KDM6B. Consequently, KDM6B further activates key osteogenic genes such as *BMP2* and *HOXC6* by removing H3K27me3, thus resulting in MSC osteogenic lineage specification, which may be the partial epigenetic mechanism of estrogen in the treatment of PMOP in the clinic [56]. Calcitriol, namely 1,25-dihydroxyvitamin D_3_ (1,25(OH)_2_D_3_), the active form of vitamin D, can be used as an adjuvant for the treatment of OP to promote calcium absorption. Mechanistically, 1,25(OH)_2_D_3_ induces the expression of EZH2 to repress the transcription of *p16*^*Ink4a*^ by trimethylating H3K27, which inhibits senescence of BM-MSCs and prevents age-related OP [15]. Therefore, EZH2 and KDM6B modulate the fate of BM-MSCs by regulating H3K27 methylation on target gene promoters and are potential therapeutic targets for OP.

In addition, mesoporous bioactive glass scaffolds containing boron (B-MBG) can induce SETD7-catalyzed H3K4 trimethylation and activate the Wnt/β-catenin pathway to promote bone regeneration in OVX rats [72]. However, the underlying mechanisms remain to be explored. Pargyline, an MAO and LSD1 inhibitor, can also rescue the osteogenic differentiation ability of BM-MSCs in aged or OVX mice by enhancing H3K4me2 at the promoters of osteogenic genes (e.g., *Runx2* and *Bglap*) [73]. Collectively, the methylase SETD7 and demethylase LSD1 dynamically modulate H3K4 methylation and regulate the osteogenic differentiation of BM-MSCs, indicating that they may be potential targets for age-related bone loss.

It is well-known that mechanical forces are indispensable for bone homeostasis and that loss of mechanical stimulation can cause disuse OP [1]. Mechanical stimulation induces osteogenic differentiation of BM-MSCs by downregulating HDAC1 expression, increasing H3 acetylation and activating pro-osteogenic JAG1-Notch signaling, and ultimately contributes to fracture healing [109]. MI192, a selective inhibitor of HDAC2 and HDAC3, can enhance the osteogenic capacity of human BM-MSCs in vitro and in mice by regulating epigenetic reprogramming [110]. Notably, nicotinamide mononucleotide (NMN) can also promote osteogenesis via the SIRT1-associated signaling pathway in aged mice [111]. However, the underlying mechanisms remain to be explored. Collectively, some HDACs and SIRT1 are also potential targets for the treatment of bone diseases such as OP by bone anabolic strategies.

## Conclusion and future perspectives

In summary, we have introduced the histone modifications and related regulatory enzymes that are implicated in fate determination of BM-MSCs during skeletal ageing. Accumulating evidence indicates that methylation at H3K27, H3K9, H3K4 and H3K36 on the promoters of osteogenic, adipogenic or senescence-associated genes closely regulates the lineage commitment and the senescent process of BM-MSCs [14, 15, 18–21, 28, 29, 47, 50, 52, 54, 55, 58, 61, 62, 64–66, 68, 70, 73–75]. In addition, acetylation of H3K9, H3K14 and H3K18 on pro-osteogenic genes, and H3K27ac on anti-osteogenic genes are tightly regulated by KATs, HDACs and SIRTs [85, 86, 88, 91–93, 98–101]. GCN5 and PCAF, both belong to the GNATs subfamily of KATs, promote osteogenic differentiation of BM-MSCs [85, 86], whereas HDAC6, 9, 11, and SIRT1 inhibit BM-MSC osteogenesis through remodeling histone deacetylation [91–94].

Although tremendous progress has been made, some issues still require further exploration. For example, causes leading to changes in histone modifications and their regulatory enzymes in the early stages of skeletal ageing remain elusive [43, 112–114]. Metabolic, nutritional, and inflammatory balances are important to the health of BM-MSCs and bone [115–118]. The disruption of these balances may affect histone modifications and enzymes, which is still less understood [119–121]. Moreover, there is extensive crosstalk among histone modifications [122]. How these protein modification interactions are involved in the maintenance of bone homeostasis remains unknown [114, 123]. Most importantly, as potential targets for treating bone diseases, the safety and efficacy of targeting histone modification enzymes require further clinical research.

## Data Availability

Not applicable.

## Abbreviations

ACE: Angiotensin-converting enzyme
α-KG: Alpha-ketoglutarate
ASH1L: Absent, small, or homeotic disc1 like
ATG7: Autophagy related protein 7
BECN1: Beclin-1
BGLAP: Osteocalcin
BMAT: Bone marrow adipose tissue
B-MBG: Mesoporous bioactive glass scaffolds containing boron
BMI1: Moloney murine leukemia virus insertion site 1
BM-MSCs: Bone marrow mesenchymal stem cells
BMP: Bone morphogenetic protein
BMPR1B: Bone morphogenetic protein receptor type 1B
CARM1: Coactivator-associated arginine methyltransferase 1
CCND1: G1/S-specific cyclin-D1
CDKN1A: Cyclin-dependent kinase inhibitor 1a
C/EBPα: CCAAT element binding protein alpha
CREB: CAMP-response element binding protein
DDR2: Discoidin domain receptor 2
DLX5: Distal-less homeobox 5
DOT1L: Disruptor of telomeric silencing 1-like
EZH2: Enhancer of zeste homolog 2
FOXO: Forkhead box O
GCN5: General control nonderepressible 5
GIOP: Glucocorticoid-induced osteoporosis
GNATs: General control of amino acid synthesis 5-related N-acetyltransferases
HDACs: Zn^2+^-dependent histone deacetylases
HDMs: Histone demethylases
HIF1α: Hypoxia-inducible factor 1-alpha
HMTs: Histone methyltransferases
HOXA10: Homeobox protein A10
HOXC6-1: Homeobox domain-containing protein 6 isoform 1
HSD11B2: 11-Beta-hydroxysteroid dehydrogenase type 2
IGF1: Insulin-like growth factor-1
JAG1: Jagged 1
JHDMs: JmjC domain-containing histone demethylases
JMJD3: Jumonji domain containing-3
KATs: Lysine acetyltransferases
KDACs: Lysine deacetylases
KDMs: Lysine demethylases
KMTs: Lysine methyltransferases
LBP: Lipopolysaccharide-binding protein
LC3a/b: Light chain 3 alpha/beta
LSD1: Lysine specific demethylase 1
MAO: Monoamine oxidase
MEF2A: Myocyte-specific enhancer factor 2A
MSCs: Mesenchymal stem cells
NAMPT: Nicotinamide phosphoribosyltransferase
NAP1L2: Nucleosome assembly protein 1-like 2
NCAPD2: Chromosome-associated protein D2
NCAPG2: Chromosome-associated protein G2
NMN: Nicotinamide mononucleotide
NO66: Nucleolar protein 66
NSD1: Nuclear receptor binding SET domain protein 1
OCT4: Octamer-binding protein 4
OP: Osteoporosis
OSX/SP7: Osterix/specificity protein 7
OVX: Ovariectomy
PAX3: Paired box 3
PCAF: P300/CBP-associated factor
PMOP: Postmenopausal osteoporosis
PPARγ: Peroxisome proliferator-activated receptor gamma
PRMT: Protein arginine methyltransferase
RBM6: RNA binding motif protein 6
RBP2: Retinol-binding protein 2
RUNX2: Runt-related transcription factor 2
SFRP: Secreted frizzled-related protein
SIRT: Sirtuin
SOX9: SRY-related HMG-box 9
TCA: Tricarboxylic acid
TCF7: Transcription factor 7
TSS: Transcription start site
UTX: Ubiquitously transcribed X-chromosome tetratricopeptide repeat protein
UTY: Y-chromosome homolog of Utx
VEGF: Vascular endothelial growth factor

## References

[1] Wang L, You X, Zhang L, Zhang C, Zou W (2022). Mechanical regulation of bone remodeling. Bone Res.

[2] Yu B, Wang CY (2016). Osteoporosis: the result of an ‘aged’ bone microenvironment. Trends Mol Med.

[3] NIH Consensus Development Panel on Osteoporosis Prevention D, and Therapy (2001). Osteoporosis prevention, diagnosis, and therapy. JAMA.

[4] Shuster S (2020). Osteoporosis, like skin ageing, is caused by collagen loss which is reversible. J R Soc Med.

[5] Jin J (2018). Screening for osteoporosis to prevent fractures. JAMA.

[6] Fazeli PK, Klibanski A (2018). Effects of anorexia nervosa on bone metabolism. Endocr Rev.

[7] Petermann-Rocha F, Ferguson LD, Gray SR, Rodriguez-Gomez I, Sattar N, Siebert S (2021). Association of sarcopenia with incident osteoporosis: a prospective study of 168,682 UK biobank participants. J Cachexia Sarcopenia Muscle.

[8] Ensrud KE, Kats AM, Boyd CM, Diem SJ, Schousboe JT, Taylor BC (2019). Association of disease definition, comorbidity burden, and prognosis with hip fracture probability among late-life women. JAMA Intern Med.

[9] Almeida M, Laurent MR, Dubois V, Claessens F, O’Brien CA, Bouillon R (2017). Estrogens and androgens in skeletal physiology and pathophysiology. Physiol Rev.

[10] Manolagas SC (2010). From estrogen-centric to aging and oxidative stress: a revised perspective of the pathogenesis of osteoporosis. Endocr Rev.

[11] Chen Q, Shou P, Zheng C, Jiang M, Cao G, Yang Q (2016). Fate decision of mesenchymal stem cells: adipocytes or osteoblasts?. Cell Death Differ.

[12] Li H, Liu P, Xu S, Li Y, Dekker JD, Li B (2017). FOXP1 controls mesenchymal stem cell commitment and senescence during skeletal aging. J Clin Invest.

[13] Fafian-Labora J, Morente-Lopez M, Sanchez-Dopico MJ, Arntz OJ, van de Loo FAJ, De Toro J (2020). Influence of mesenchymal stem cell-derived extracellular vesicles in vitro and their role in ageing. Stem Cell Res Ther.

[14] Li C, Chai Y, Wang L, Gao B, Chen H, Gao P (2017). Programmed cell senescence in skeleton during late puberty. Nat Commun.

[15] Yang R, Chen J, Zhang J, Qin R, Wang R, Qiu Y (2020). 1,25-Dihydroxyvitamin D protects against age-related osteoporosis by a novel VDR-Ezh2-p16 signal axis. Aging Cell.

[16] Li Y, Lu L, Xie Y, Chen X, Tian L, Liang Y (2020). Interleukin-6 knockout inhibits senescence of bone mesenchymal stem cells in high-fat diet-induced bone loss. Front Endocrinol.

[17] Tencerova M, Frost M, Figeac F, Nielsen TK, Ali D, Lauterlein JL (2019). Obesity-associated hypermetabolism and accelerated senescence of bone marrow stromal stem cells suggest a potential mechanism for bone fragility. Cell Rep.

[18] Deng P, Yuan Q, Cheng Y, Li J, Liu Z, Liu Y (2021). Loss of KDM4B exacerbates bone-fat imbalance and mesenchymal stromal cell exhaustion in skeletal aging. Cell Stem Cell.

[19] Su X, Zhang H, Lei F, Wang R, Lin T, Liao L (2022). Epigenetic therapy attenuates oxidative stress in BMSCs during ageing. J Cell Mol Med.

[20] Wang Y, Deng P, Liu Y, Wu Y, Chen Y, Guo Y (2020). Alpha-ketoglutarate ameliorates age-related osteoporosis via regulating histone methylations. Nat Commun.

[21] Xie Y, Han N, Li F, Wang L, Liu G, Hu M (2022). Melatonin enhances osteoblastogenesis of senescent bone marrow stromal cells through NSD2-mediated chromatin remodelling. Clin Transl Med.

[22] Gomathi K, Akshaya N, Srinaath N, Rohini M, Selvamurugan N (2021). Histone acetyl transferases and their epigenetic impact on bone remodeling. Int J Biol Macromol.

[23] Curtis EM, Fuggle NR, Cooper C, Harvey NC (2022). Epigenetic regulation of bone mass. Best Pract Res Clin Endocrinol Metab.

[24] Sharma G, Sultana A, Abdullah KM, Pothuraju R, Nasser MW, Batra SK (2022). Epigenetic regulation of bone remodeling and bone metastasis. Semin Cell Dev Biol.

[25] Sun P, Huang T, Huang C, Wang Y, Tang D (2022). Role of histone modification in the occurrence and development of osteoporosis. Front Endocrinol.

[26] Ren R, Ocampo A, Liu GH, Izpisua Belmonte JC (2017). Regulation of Stem cell aging by metabolism and epigenetics. Cell Metab.

[27] Pal S, Tyler JK (2016). Epigenetics and aging. Sci Adv.

[28] Jing H, Liao L, An Y, Su X, Liu S, Shuai Y (2016). Suppression of EZH2 prevents the shift of osteoporotic MSC fate to adipocyte and enhances bone formation during osteoporosis. Mol Ther.

[29] Wang L, Niu N, Li L, Shao R, Ouyang H, Zou W (2018). H3K36 trimethylation mediated by SETD2 regulates the fate of bone marrow mesenchymal stem cells. PLoS Biol.

[30] Liu C, Xiong Q, Li Q, Lin W, Jiang S, Zhang D (2022). CHD7 regulates bone-fat balance by suppressing PPAR-gamma signaling. Nat Commun.

[31] Kouzarides T (2007). Chromatin modifications and their function. Cell.

[32] Lawrence M, Daujat S, Schneider R (2016). Lateral thinking: how histone modifications regulate gene expression. Trends Genet.

[33] Yun M, Wu J, Workman JL, Li B (2011). Readers of histone modifications. Cell Res.

[34] Strahl BD, Allis CD (2000). The language of covalent histone modifications. Nature.

[35] Guccione E, Richard S (2019). The regulation, functions and clinical relevance of arginine methylation. Nat Rev Mol Cell Biol.

[36] Kaniskan HU, Martini ML, Jin J (2018). Inhibitors of protein methyltransferases and demethylases. Chem Rev.

[37] Jambhekar A, Dhall A, Shi Y (2019). Roles and regulation of histone methylation in animal development. Nat Rev Mol Cell Biol.

[38] Rivenbark AG, Strahl BD (2007). Molecular biology. Unlocking cell fate. Science.

[39] Pan MR, Hsu MC, Chen LT, Hung WC (2018). Orchestration of H3K27 methylation: mechanisms and therapeutic implication. Cell Mol Life Sci.

[40] Choudhary C, Weinert BT, Nishida Y, Verdin E, Mann M (2014). The growing landscape of lysine acetylation links metabolism and cell signalling. Nat Rev Mol Cell Biol.

[41] Allis CD, Jenuwein T (2016). The molecular hallmarks of epigenetic control. Nat Rev Genet.

[42] Shvedunova M, Akhtar A (2022). Modulation of cellular processes by histone and non-histone protein acetylation. Nat Rev Mol Cell Biol.

[43] Deng P, Chen QM, Hong C, Wang CY (2015). Histone methyltransferases and demethylases: regulators in balancing osteogenic and adipogenic differentiation of mesenchymal stem cells. Int J Oral Sci.

[44] Saidi N, Ghalavand M, Hashemzadeh MS, Dorostkar R, Mohammadi H, Mahdian-Shakib A (2017). Dynamic changes of epigenetic signatures during chondrogenic and adipogenic differentiation of mesenchymal stem cells. Biomed Pharmacother.

[45] Killaars AR, Walker CJ, Anseth KS (2020). Nuclear mechanosensing controls MSC osteogenic potential through HDAC epigenetic remodeling. Proc Natl Acad Sci U S A.

[46] Wei Y, Chen YH, Li LY, Lang J, Yeh SP, Shi B (2011). CDK1-dependent phosphorylation of EZH2 suppresses methylation of H3K27 and promotes osteogenic differentiation of human mesenchymal stem cells. Nat Cell Biol.

[47] Hemming S, Cakouros D, Isenmann S, Cooper L, Menicanin D, Zannettino A (2014). EZH2 and KDM6A act as an epigenetic switch to regulate mesenchymal stem cell lineage specification. Stem Cells.

[48] Wang L, Jin Q, Lee JE, Su IH, Ge K (2010). Histone H3K27 methyltransferase Ezh2 represses Wnt genes to facilitate adipogenesis. Proc Natl Acad Sci U S A.

[49] Dudakovic A, Camilleri ET, Xu F, Riester SM, McGee-Lawrence ME, Bradley EW (2015). Epigenetic control of skeletal development by the histone methyltransferase Ezh2. J Biol Chem.

[50] Hemming S, Cakouros D, Codrington J, Vandyke K, Arthur A, Zannettino A (2017). EZH2 deletion in early mesenchyme compromises postnatal bone microarchitecture and structural integrity and accelerates remodeling. FASEB J.

[51] Swigut T, Wysocka J (2007). H3K27 demethylases, at long last. Cell.

[52] Shuai Y, Yang R, Mu R, Yu Y, Rong L, Jin L (2019). MiR-199a-3p mediates the adipogenic differentiation of bone marrow-derived mesenchymal stem cells by regulating KDM6A/WNT signaling. Life Sci.

[53] Wang FS, Lian WS, Lee MS, Weng WT, Huang YH, Chen YS (2017). Histone demethylase UTX counteracts glucocorticoid deregulation of osteogenesis by modulating histone-dependent and -independent pathways. J Mol Med (Berl).

[54] Ye L, Fan Z, Yu B, Chang J, Al Hezaimi K, Zhou X (2012). Histone demethylases KDM4B and KDM6B promotes osteogenic differentiation of human MSCs. Cell Stem Cell.

[55] Behera J, Ison J, Rai H, Tyagi N (2021). Allyl sulfide promotes osteoblast differentiation and bone density via reducing mitochondrial DNA release mediated Kdm6b/H3K27me3 epigenetic mechanism. Biochem Biophys Res Commun.

[56] Liu Z, Lee HL, Suh JS, Deng P, Lee CR, Bezouglaia O (2022). The ERalpha/KDM6B regulatory axis modulates osteogenic differentiation in human mesenchymal stem cells. Bone Res.

[57] Huang C, Xiang Y, Wang Y, Li X, Xu L, Zhu Z (2010). Dual-specificity histone demethylase KIAA1718 (KDM7A) regulates neural differentiation through FGF4. Cell Res.

[58] Yang X, Wang G, Wang Y, Zhou J, Yuan H, Li X (2019). Histone demethylase KDM7A reciprocally regulates adipogenic and osteogenic differentiation via regulation of C/EBPalpha and canonical Wnt signalling. J Cell Mol Med.

[59] Shahmirzadi AA, Edgar D, Liao CY, Hsu YM, Lucanic M, Shahmirzadi AA (2020). Alpha-ketoglutarate, an endogenous metabolite, extends lifespan and compresses morbidity in aging mice. Cell Metab.

[60] Bayliak MM, Lushchak VI (2021). Pleiotropic effects of alpha-ketoglutarate as a potential anti-ageing agent. Ageing Res Rev.

[61] Cakouros D, Isenmann S, Cooper L, Zannettino A, Anderson P, Glackin C (2012). Twist-1 induces Ezh2 recruitment regulating histone methylation along the Ink4A/Arf locus in mesenchymal stem cells. Mol Cell Biol.

[62] Hu T, Kitano A, Luu V, Dawson B, Hoegenauer KA, Lee BH (2019). Bmi1 suppresses adipogenesis in the hematopoietic stem cell niche. Stem Cell Rep.

[63] Shi Y, Lan F, Matson C, Mulligan P, Whetstine JR, Cole PA (2004). Histone demethylation mediated by the nuclear amine oxidase homolog LSD1. Cell.

[64] Zhang Y, Ma C, Liu X, Wu Z, Yan P, Ma N (2015). Epigenetic landscape in PPARgamma2 in the enhancement of adipogenesis of mouse osteoporotic bone marrow stromal cell. Biochim Biophys Acta.

[65] Qi Q, Wang Y, Wang X, Yang J, Xie Y, Zhou J (2020). Histone demethylase KDM4A regulates adipogenic and osteogenic differentiation via epigenetic regulation of C/EBPalpha and canonical Wnt signaling. Cell Mol Life Sci.

[66] Qin G, Li Y, Wang H, Yang J, Chen Q, Tang H (2020). Lysine-specific demethylase 4A regulates osteogenic differentiation via regulating the binding ability of H3K9me3 with the promoters of Runx2, osterix and osteocalcin. J Biomed Nanotechnol.

[67] Nakayama J, Rice JC, Strahl BD, Allis CD, Grewal SI (2001). Role of histone H3 lysine 9 methylation in epigenetic control of heterochromatin assembly. Science.

[68] Huang B, Wang B, Yuk-Wai Lee W, Pong UK, Leung KT, Li X (2019). KDM3A and KDM4C regulate mesenchymal stromal cell senescence and bone aging via condensin-mediated heterochromatin reorganization. iScience.

[69] Bi W, Deng JM, Zhang Z, Behringer RR, de Crombrugghe B (1999). Sox9 is required for cartilage formation. Nat Genet.

[70] Yin B, Yu F, Wang C, Li B, Liu M, Ye L (2019). Epigenetic control of mesenchymal stem cell fate decision via histone methyltransferase Ash1l. Stem Cells.

[71] Gu Y, Wang Y, Wang X, Gao L, Yu W, Dong WF (2017). Opposite effects of SET7/9 on apoptosis of human acute myeloid leukemia cells and lung cancer cells. J Cancer.

[72] Yin C, Jia X, Miron RJ, Long Q, Xu H, Wei Y (2018). Setd7 and its contribution to Boron-induced bone regeneration in Boron-mesoporous bioactive glass scaffolds. Acta Biomater.

[73] Lv L, Ge W, Liu Y, Lai G, Liu H, Li W (2016). Lysine-specific demethylase 1 inhibitor rescues the osteogenic ability of mesenchymal stem cells under osteoporotic conditions by modulating H3K4 methylation. Bone Res.

[74] Sun J, Ermann J, Niu N, Yan G, Yang Y, Shi Y (2018). Histone demethylase LSD1 regulates bone mass by controlling WNT7B and BMP2 signaling in osteoblasts. Bone Res.

[75] Wang C, Wang J, Li J, Hu G, Shan S, Li Q (2016). KDM5A controls bone morphogenic protein 2-induced osteogenic differentiation of bone mesenchymal stem cells during osteoporosis. Cell Death Dis.

[76] Rojas A, Aguilar R, Henriquez B, Lian JB, Stein JL, Stein GS (2015). Epigenetic control of the bone-master Runx2 gene during osteoblast-lineage commitment by the histone demethylase JARID1B/KDM5B. J Biol Chem.

[77] Li W, Tian W, Yuan G, Deng P, Sengupta D, Cheng Z (2021). Molecular basis of nucleosomal H3K36 methylation by NSD methyltransferases. Nature.

[78] Shao R, Zhang Z, Xu Z, Ouyang H, Wang L, Ouyang H (2021). H3K36 methyltransferase NSD1 regulates chondrocyte differentiation for skeletal development and fracture repair. Bone Res.

[79] Gavalda-Navarro A, Moreno-Navarrete JM, Quesada-Lopez T, Cairo M, Giralt M, Fernandez-Real JM (2016). Lipopolysaccharide-binding protein is a negative regulator of adipose tissue browning in mice and humans. Diabetologia.

[80] Chen Q, Zhang L, de Crombrugghe B, Krahe R (2015). Mesenchyme-specific overexpression of nucleolar protein 66 in mice inhibits skeletal growth and bone formation. FASEB J.

[81] Chen Q, Sinha K, Deng JM, Yasuda H, Krahe R, Behringer RR (2015). Mesenchymal deletion of histone demethylase NO66 in mice promotes bone formation. J Bone Miner Res.

[82] Li P, Ge J, Li H (2020). Lysine acetyltransferases and lysine deacetylases as targets for cardiovascular disease. Nat Rev Cardiol.

[83] Menzies KJ, Zhang H, Katsyuba E, Auwerx J (2016). Protein acetylation in metabolism–metabolites and cofactors. Nat Rev Endocrinol.

[84] Zhao QH, Wang SG, Liu SX, Li JP, Zhang YX, Sun ZY (2013). PPARgamma forms a bridge between DNA methylation and histone acetylation at the C/EBPalpha gene promoter to regulate the balance between osteogenesis and adipogenesis of bone marrow stromal cells. FEBS J.

[85] Jing H, Su X, Gao B, Shuai Y, Chen J, Deng Z (2018). Epigenetic inhibition of Wnt pathway suppresses osteogenic differentiation of BMSCs during osteoporosis. Cell Death Dis.

[86] Zhang P, Liu Y, Jin C, Zhang M, Lv L, Zhang X (2016). Histone H3K9 acetyltransferase PCAF is essential for osteogenic differentiation through bone morphogenetic protein signaling and may be involved in osteoporosis. Stem Cells.

[87] Ling M, Huang P, Islam S, Heruth DP, Li X, Zhang LQ (2017). Epigenetic regulation of Runx2 transcription and osteoblast differentiation by nicotinamide phosphoribosyltransferase. Cell Biosci.

[88] Jing H, Liao L, Su X, Shuai Y, Zhang X, Deng Z (2017). Declining histone acetyltransferase GCN5 represses BMSC-mediated angiogenesis during osteoporosis. FASEB J.

[89] Zhang P, Liu Y, Jin C, Zhang M, Tang F, Zhou Y (2016). Histone acetyltransferase GCN5 regulates osteogenic differentiation of mesenchymal stem cells by inhibiting NF-kappaB. J Bone Miner Res.

[90] Nacarelli T, Lau L, Fukumoto T, Zundell J, Fatkhutdinov N, Wu S (2019). NAD(+) metabolism governs the proinflammatory senescence-associated secretome. Nat Cell Biol.

[91] Ma C, Gao J, Liang J, Dai W, Wang Z, Xia M (2021). HDAC6 inactivates Runx2 promoter to block osteogenesis of bone marrow stromal cells in age-related bone loss of mice. Stem Cell Res Ther.

[92] Zhang L, Qi M, Chen J, Zhao J, Li L, Hu J (2020). Impaired autophagy triggered by HDAC9 in mesenchymal stem cells accelerates bone mass loss. Stem Cell Res Ther.

[93] Wu Z, Wen Y, Xiao H, Zhu J, Li B, Shangguan Y (2022). 11beta-Hydroxysteroid dehydrogenase 2: a key mediator of high susceptibility to osteoporosis in offspring after prenatal dexamethasone exposure. Pharmacol Res.

[94] Hu M, Xing L, Zhang L, Liu F, Wang S, Xie Y (2022). NAP1L2 drives mesenchymal stem cell senescence and suppresses osteogenic differentiation. Aging Cell.

[95] Shangguan Y, Wen Y, Tan Y, Qin J, Jiang H, Magdalou J (2018). Intrauterine programming of glucocorticoid-insulin-like growth factor-1 axis-mediated developmental origin of osteoporosis susceptibility in female offspring rats with prenatal caffeine exposure. Am J Pathol.

[96] Bachagol D, Joseph GS, Ellur G, Patel K, Aruna P, Mittal M (2018). Stimulation of liver IGF-1 expression promotes peak bone mass achievement in growing rats: a study with pomegranate seed oil. J Nutr Biochem.

[97] Weinert BT, Narita T, Satpathy S, Srinivasan B, Hansen BK, Scholz C (2018). Time-resolved analysis reveals rapid dynamics and broad scope of the CBP/p300 acetylome. Cell.

[98] Xiao H, Wen Y, Pan Z, Shangguan Y, Qin J, Tan Y (2018). Increased H3K27ac level of ACE mediates the intergenerational effect of low peak bone mass induced by prenatal dexamethasone exposure in male offspring rats. Cell Death Dis.

[99] Wu Z, Pan Z, Wen Y, Xiao H, Shangguan Y, Wang H (2020). Egr1/p300/ACE signal mediates postnatal osteopenia in female rat offspring induced by prenatal ethanol exposure. Food Chem Toxicol.

[100] Chen JR, Lazarenko OP, Zhao H, Alund AW, Shankar K (2018). Maternal obesity impairs skeletal development in adult offspring. J Endocrinol.

[101] Liu H, Hu L, Yu G, Yang H, Cao Y, Wang S (2021). LncRNA, PLXDC2-OT promoted the osteogenesis potentials of MSCs by inhibiting the deacetylation function of RBM6/SIRT7 complex and OSX specific isoform. Stem Cells.

[102] Chen EEM, Zhang W, Ye CCY, Gao X, Jiang LLJ, Zhao TTF (2017). Knockdown of SIRT7 enhances the osteogenic differentiation of human bone marrow mesenchymal stem cells partly via activation of the Wnt/beta-catenin signaling pathway. Cell Death Dis.

[103] Min Z, Xiaomeng L, Zheng L, Yangge D, Xuejiao L, Longwei L (2019). Asymmetrical methyltransferase PRMT3 regulates human mesenchymal stem cell osteogenesis via miR-3648. Cell Death Dis.

[104] Jo J, Song H, Park SG, Lee SH, Ko JJ, Park JH (2012). Regulation of differentiation potential of human mesenchymal stem cells by intracytoplasmic delivery of coactivator-associated arginine methyltransferase 1 protein using cell-penetrating peptide. Stem Cells.

[105] Xu Z, Wu W, Shen F, Yu Y, Wang Y, Xiang C (2019). Histone arginine methylation-mediated epigenetic regulation of discoidin domain receptor 2 controls the senescence of human bone marrow mesenchymal stem cells. Stem Cells Int.

[106] Holdgate GA, Bardelle C, Lanne A, Read J, O'Donovan DH, Smith JM (2022). Drug discovery for epigenetics targets. Drug Discov Today.

[107] Galvan ML, Paradise CR, Kubrova E, Jerez S, Khani F, Thaler R (2021). Multiple pharmacological inhibitors targeting the epigenetic suppressor enhancer of zeste homolog 2 (Ezh2) accelerate osteoblast differentiation. Bone.

[108] Dudakovic A, Camilleri ET, Riester SM, Paradise CR, Gluscevic M, O'Toole TM (2016). Enhancer of zeste homolog 2 inhibition stimulates bone formation and mitigates bone loss caused by ovariectomy in skeletally mature mice. J Biol Chem.

[109] Wang J, Wang CD, Zhang N, Tong WX, Zhang YF, Shan SZ (2016). Mechanical stimulation orchestrates the osteogenic differentiation of human bone marrow stromal cells by regulating HDAC1. Cell Death Dis.

[110] Man K, Mekhileri NV, Lim KS, Jiang LH, Woodfield TBF, Yang XB (2021). MI192 induced epigenetic reprogramming enhances the therapeutic efficacy of human bone marrows stromal cells for bone regeneration. Bone.

[111] Song J, Li J, Yang F, Ning G, Zhen L, Wu L (2019). Nicotinamide mononucleotide promotes osteogenesis and reduces adipogenesis by regulating mesenchymal stromal cells via the SIRT1 pathway in aged bone marrow. Cell Death Dis.

[112] Clarke J (2021). Ageing stem cells hold the key to age-related bone degeneration. Nat Rev Rheumatol.

[113] Seeman E (2002). Pathogenesis of bone fragility in women and men. Lancet.

[114] Sui BD, Zheng CX, Li M, Jin Y, Hu CH (2020). Epigenetic regulation of mesenchymal stem cell homeostasis. Trends Cell Biol.

[115] Loeffler J, Duda GN, Sass FA, Dienelt A (2018). The metabolic microenvironment steers bone tissue regeneration. Trends Endocrinol Metab.

[116] Hayashi M, Nakashima T, Yoshimura N, Okamoto K, Tanaka S, Takayanagi H (2019). Autoregulation of osteocyte Sema3A orchestrates estrogen action and counteracts bone aging. Cell Metab.

[117] Rizzoli R, Biver E, Brennan-Speranza TC (2021). Nutritional intake and bone health. Lancet Diabetes Endocrinol.

[118] Josephson AM, Bradaschia-Correa V, Lee S, Leclerc K, Patel KS, Muinos Lopez E (2019). Age-related inflammation triggers skeletal stem/progenitor cell dysfunction. Proc Natl Acad Sci U S A.

[119] Tarazona OA, Pourquie O (2020). Exploring the influence of cell metabolism on cell fate through protein post-translational modifications. Dev Cell.

[120] Cai S, Quan S, Yang G, Chen M, Ye Q, Wang G (2021). Nutritional status impacts epigenetic regulation in early embryo development: a scoping review. Adv Nutr.

[121] Evans LW, Stratton MS, Ferguson BS (2020). Dietary natural products as epigenetic modifiers in aging-associated inflammation and disease. Nat Prod Rep.

[122] Suganuma T, Workman JL (2008). Crosstalk among histone modifications. Cell.

[123] Ren J, Huang D, Li R, Wang W, Zhou C (2020). Control of mesenchymal stem cell biology by histone modifications. Cell Biosci.

